# Gender differences and individual, household, and workplace characteristics: Regional geographies of extended working lives

**DOI:** 10.1002/psp.2213

**Published:** 2018-11-21

**Authors:** Nicola Shelton, Jenny Head, Ewan Carr, Paola Zaninotto, Gareth Hagger‐Johnson, Emily Murray

**Affiliations:** ^1^ Epidemiology and Public Health UCL London UK; ^2^ Institute of Psychiatry King's College London UK

**Keywords:** later life work, longitudinal study, retirement, unemployment, work exit

## Abstract

Increasing labour market participation among older workers is embedded in government policy in the United Kingdom and many other industrialised countries with rises in the state pension age in response to increasing life expectancy. Despite this, many workers stop working before state pension age with around a 20% reduction in the proportion of adults in work between ages 50 and 60 in 2011 in England and Wales. This paper considers the risk of remaining in work by region and gender between 2001 and 2011 for adults aged 40–49 in 2001. Men had significantly higher risk of extended working in the East Midlands (1.4×) East of England (1.5×), South East (1.6×), and South West (1.6×) compared with the North East. Women in all regions apart from London and Wales had significantly higher risk of extended working compared with the North East: ranging from 1.15 times in the North West and West Midlands to 1.6 times in the South West. Adjustment for nonemployment‐related socio‐economic status, housing tenure, qualifications, and car ownership, and employment status in 2001 attenuated all significant regional differences in extended working in men and in women in most regions. Workplace characteristics attenuated most of the remaining regional differences in women: women working in larger employers in 2001 or working at distances of 200 km or more, abroad or from home, had lower risk of remaining in work, whereas access to a car and higher working hours increased risk. Policies to increase qualifications and skills among older adults are recommended.

## 1 INTRODUCTION

Government policy in the United Kingdom and many other industrialised countries is to raise state pension age in response to the financial challenges of increasing life expectancy and population ageing. Increasing labour market participation among older workers is expected to deliver a “double dividend” with reduced demands on health and social care services and a contribution to the economy in terms of paid work (Chandler & Tetlow, 2014). Extending working lives (EWL) beyond traditional retirement ages (generally some years below state pension age) can also have individual financial and health benefits. Given a good work environment, choosing to remain in work may have positive benefits such as maintaining good health and functioning and providing a sense of purpose (Maimaris, Hogan, & Lock, 2010).

Employment rates of men and women aged 50 and over have been rising since the mid‐1990s when employment in the over 65s was around 5% and in those aged 50–64 years around 65% (though previously, rates had been higher in men in the 1960s and 1970s; Crawford & Tetlow, 2010). In 2011, U.K. employment rates (based on data from the Labour Force Survey) were 80% among people aged 50–54 and 44% for those aged 60–64 (Department for Work and Pensions, 2011). Multiple “push and pull” factors that operate inside and outside the workplace, at the individual and contextual levels, are associated with not being in work at older ages. These include positive financial incentives or negative financial circumstances (such as inadequate pension provision), working for enjoyment and self‐esteem, or may be based on peer norms (Warr, Butcher, Robertson, & Callinan, 2004). Encouraging higher employment rates among the over 50s will therefore depend on both incentivising work and removing the barriers to participation. Retaining older people in the workforce in their current employment even up to the state pension age may be challenging where early retirement dividends are paid by company pensions or (voluntary) redundancy payments are offered that require exiting from the workforce. Private pensions are usually accessible from age 55 (The Pensions Advisory Service, 2018) meaning there is a big gap in the age that those with company pensions may be able to afford to retire comfortably compared with those relying on the state pension.

The Department for Work and Pensions (2012) identified subpopulations that were considered to be both “high risk/vulnerable groups” and “more receptive groups” for extended working stating that “The capacity to identify employment sectors, geographical regions or locales where concentrations of these sub‐populations exist offers further potential to concentrate resources to maximal effect.” Their expectation was that those at high risk of unemployment were potentially “high resistance” and they argued that it may be more viable to concentrate resources on subpopulations that are more receptive to extension. This policy implies that there is free choice in decisions about extended working and that unemployment in later life is due to resistance to working, not unequal availability of employment opportunities.

The availability of suitable employment opportunities varies by region: Employment rates based on Labour Force Survey data for people aged 50–64 varied from 69.6% in the South East to 58.5% in the North East in 2011 (Department for Work and Pensions, 2011). The North East also had fewer professional and skilled trade occupations skills shortage vacancies (Round, 2016). Previous studies have shown associations between local unemployment and deprivation levels and retirement rates in France (Korsu & Wenglenski, 2010), Norway (Krokstad, Magnus, Skrondal, & Westin, 2004; Reime & Claussen, 2013), and Great Britain (Fieldhouse & Gould, 1998). More recently, Murray et al. (2016) have shown that labour market exit was related to local area unemployment in older adults in England and Wales in 2011 and argued that uniform national and occupational postponement of pensionable age may be inappropriate because retention of older persons in the workforce is not distributed equally across geographical areas. The State Pension Age Review rejected the suggestion that pension age should not be universal addressing regional inequalities in life expectancy, arguing that there was no evidence submitted for workable ways for State Pension Age to address this (Cridland, 2017).

Older women have traditionally had shorter working lives than men due to absences from the workforce for child rearing and historically due to barriers with a lack of legislation and support for them to remain in the workplace once they are married or had children. Additionally, until 2018, state pension ages in the United Kingdom have been lower for women (The Pensions Advisory Service, 2018). Changes in legislation in the 1970s promoting equal pay (enacted 1970 and implemented 1975; legislation.gov.uk, 2016) and protecting women's right to work in 1975 (legislation.gov.uk, 2018) and increasing financial support for working parents have helped expand the number of women in the workforce overall. In the United Kingdom, the female employment rate had increased from 52.8% in the first quarter of 1971 to 69.2% in the first quarter of 2016 (with a fall in the male employment rate over the same period from 92.1% to 79.3%, respectively; Office for National Statistics [ONS], 2017, 2018) though the female employment rate remains at lower level than male. Women generally retire earlier than men and were able to claim their U.K. state person at age 60 compared with men at age 65, in 2011 (Chandler & Tetlow, 2014). Increases in the State Pension Age for women to be comparable with men by 2018 (The Pensions Advisory Service, 2017) and higher proportions of women than men living alone in their midlife (Demey, Berrington, Evandrou, & Falkingham, 2013) and women's longer and increasing life expectancy at older ages (ONS, 2011) may mean that women need to rely on extended working far more than previously for later life income. In addition, older women workers report that employment helps keep them active and gives them feelings of value to society (Doyal, 2000). Conversely, many of the sectors where there have been declines in employment have been traditionally male dominated (Equal Opportunities Commission, 2005) so the regional distribution of opportunity for women extending their working lives may be very different both to that of men and to that experienced previously more generally.

Much of the wider narrative has been around women stopping work at much younger ages typically for family considerations (Ruhm, 1996). Loretto and Vickerstaff (2015) argued that the lack of attention paid to the role of gender in EWL is due to little consideration of relationships between gender and flexible working beyond the child‐caring phase of life; the prevailing tendency is to think of end of working life and retirement as gender‐neutral or following a typical male trajectory). Much of the literature discussing gender differences in employment rates focuses on gender differences in caring (e.g., Arber & Ginn, 1995) or female roles. Ruhm (1996) showed that unmarried 55‐ to 59‐year‐old men and women with some recent history of employment in the United States in 1989 had similar employment rates, whereas married men were much more likely than married women to work. Nonworking women were more likely to leave work for family‐related reasons, and men were more likely to report finances or employer factors. One early study by Rosenfeld and Perrella (1965) using data from the United States in 1963 looked at why women aged 18–64 took up and jobs and stopped working at different ages. In the 45‐ to 64‐year age group, 64% of women reported financial reasons for taking up work whereas only 18% reported personal satisfaction. Personal satisfaction as a reason for taking up work was highest in women aged 45‐64 whose husband's usual weekly income was $100 or more (26%) but only 4% in women whose husband's usually weekly income was less than $60.

There is limited literature on how and why regional differences occur in the later life labour market by gender. Sackmann and Haussermann (1994) found that western states in the Federal Republic in Germany in 1987 had lower levels of female employment than southern and northern states at all ages and higher full employment in regions with overall higher female employment rates. They described long‐term “regional cultures,” with definition of a female role (lower labour market participation) by employers and by women themselves, being more relevant than industrial sectoral structures. Similarly in Sweden, Forsberg (1998) argued for an explanation of regional differences by differing gender contracts with labour market segregation and social infrastructure influences. The idea that low rates of female labour market participation imply a different role or culture around women's work is implicit.

Previous research using the ONS Longitudinal Study (ONS‐LS) and the English Longitudinal Study of Ageing analysed factors affecting labour withdrawal including region in England and Wales in those aged 50–64 (Dini, 2010). The significant factors in decreasing labour market exit were lower level educational qualifications compared with degree (women only) and owning a house with a mortgage compared with owning outright; the significant factors in increasing labour market exit at an older age were Indian, Pakistani, and Bangladeshi ethnicities compared with White ethnicity (women only), single marital status (women only), prior intermediate routine and manual occupations compared with professional, previously being unemployed, having health problems, having no dependent children in the household, and residential location. The study included just two areas: South, East, and Midlands (reference category) compared with North of England and Wales. Area was significantly associated with extended working after adjustment for individual characteristics, with those in the North of England and Wales having lower odds of extended working. One of the notable findings of the study was that far fewer factors were significant for men compared with women, but this difference in the gender influences on extended working life has not been further considered at a lower geographical level, and it is this issue that this paper seeks to address. The disaggregation of labour market participation by gender, age group, and region is difficult to study in many individual‐level data sets due to sample size constraints.

This analysis uses data from a large, nationally representative linked census longitudinal study, the ONS‐LS to investigate, for a group of women (*n* = 33,124) and men (*n* = 31,696) aged 40–49 in 2001 (so born between 1946 and 1956) and aged 50–59 in 2011, the factors associated with their being in work in 2011 up to age 59 (below state pension age). A subsample (*n* = 25,008) was also analysed restricted to women in work in 2001 to elucidate whether regional differences in remaining in work are associated with workplace‐related characteristics as only those in work in 2001 have this information.

The paper considers the following:

- Are there regional differences in EWL in England and Wales? How do those differ for men and women?
- Which working conditions or household and individual factors promote EWL, and do they attenuate or exacerbate any regional variation?

## 2 HOW DO REGIONAL VARIATIONS DIFFER BY GENDER?

### 2.1 Data

#### 2.1.1 Study participants

The research uses data from a national longitudinal study, the ONS‐LS, which links census records and records of deaths, cancers, and births for approximately 1.1% of the population of England and Wales. Currently, data from each decennial census 1971 to 2011 inclusive are available in the ONS‐LS, along with death registrations. We used data from 2001 and 2011. New members are added to the LS if either newly born children or immigrants have the four birthdates from which the sample is drawn. This sample was chosen to reflect contemporary individuals at ages where they would be at higher risk of exiting the work force, but were not yet at compulsory retirement age, which was abolished after the date of the 2011 census when the working status data used in this study were collected.

#### 2.1.2 Outcome: Work status variables

Work status variables were collected at both the 2001 and 2011 censuses. Respondents completed a tick box of options used to determine their participation in paid work in the labour market in the week preceding each census. Working status in 2001 with those respondents considered to be “in work” (this included working, on temporary sick leave, maternity leave, holiday, or about to take up a job) was used as a risk factor for analysis for 2011 employment with workplace characteristics for subset analysis restricted to these in work in 2001. For the outcome in 2011, adults were coded as in work, not in work, or if they had died between the 2001 and 2011 censuses, they were kept in the sample and coded as dead before 2011 census date (the mortality outcome data are not shown). If the respondent ticked “no” to doing paid work in the last week, they were asked if they had ever worked and the year they had last worked.

#### 2.1.3 Area

Area was coded into former Government Office Regions with nine regions of North East (reference category), North West, Yorkshire and Humber, East Midlands, West Midlands, East of England, London, South East, South West, and Wales.

#### 2.1.4 Employment conditions

Additional characteristics of the workplace and job were also included in the subset analysis: Occupational social class was based on the Registrar General's classification (CeLSIUS, 2013), collapsed into three categories of nonmanual, manual, and other (includes armed forces and no job class identifiable). Employer size in 2001 was coded as 1–19, 20–24, 25–499, and 500 or more employees. Working hours were coded as a linear variable. Distance to workplace was coded as 0–49, 50–99, 100–199, and 200 km or more, with separate categories for worked abroad and worked from home.

#### 2.1.5 Individual and household characteristics

Demographic and socio‐economic indicators in 2001 were included as potential covariates. Demographic variables included the following: (a) age; (b) sex; (c) ethnicity, classified into five categories of White, mixed, Asian, Black, and other; (d) marital status and spousal working status combined, no spouse, spouse working, spouse not working, and spouse not working due to sickness; and (e) age of the youngest dependent child in household, no dependent children and youngest aged 0–4, aged 5–11, aged 12–15, and aged 16–18. Socio‐economic indicators included housing tenure, classified into six categories of owner, mortgaged, social rent, private rent, rent free, or other; hours of caring in last week, no care provided, 1–19 hr, 20–49 hr, and 50 or more hours; highest educational qualification coded as none, less than five GCSE/O Levels, five or more GCSE/O Levels, two A Levels or equivalent, degree and above, and other/unknown; and number of cars (treated as a linear variable). Two health indicators were assessed in the census: (a) long‐term limiting illness “a long‐term illness, health problem or disability which limits your daily activities or the work you can do” and (b) self‐rated health “over the last 12 months would you say your health has on the whole been: good, fairly good or not good?”

## 3 STATISTICAL ANALYSIS

All predictors were compared across 2011 work status categories (in paid work, not working, and died before 2011 census). The analysis used was multinomial multivariate logistic regression, with not being in work in 2011 as the reference category, compared with two categories of being in work in 2011, and dying between 2001 and 2011. The analysis was carried out in this way because a significant proportion of the sample had died within the 10‐year period and also because those that died would be generally have been in poorer health prior to death, and this would vary regionally. Men and women estimates for extended working lives were analysed separately, minimally adjusted for prior working status in 2001, and then additionally adjusted for the covariates listed above in groups until no regional variations remained. Firstly models were adjusted for working status in 2001, then socio‐economic variables, then demographic variables (ethnicity, marital status and depending children, and age), then health status and caring status, and finally workplace variables for those in work in 2001. Age of labour market exit prior to 2011 was also plotted for a larger sample who were working and aged 40–69 in 2001 to illustrate the age pattern of work exit prior to state pension age.

## 4 RESULTS

**Figure 1:**
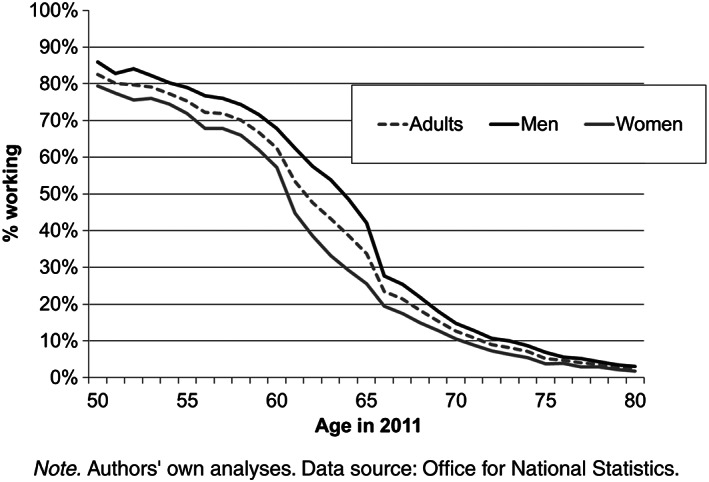
Proportion of adults aged 50 and over working in 2011, England and Wales

Figure 1 shows the proportion of adults over the age 50 and above in work in 2011 in England and Wales. A higher proportion of men compared with women are in work at all ages, but there is approximately a 20% reduction in the proportion of adults in work in both men and women between ages 50 and 60 prior to state retirement age in 2011. Notable turning points occurred around age 60 for men and women and age 50 for men. This has important implications for statistics that are usually collated in 5‐year age groups—the 60‐ to 64‐year age group will include those about to retire at age 60.

Table 1 shows the relative regional risk ratios for extended work for men. Model 1 shows the regional differences in relative risk ratios for extended working in 2011, adjusted for demographic factors in 2001 in men aged 40–49. Unadjusted ratios show that men were significantly more likely to experience extended working in the East Midlands (1.4×), East of England (1.5×), South East (1.6×), and South West (1.6×) compared with the North East (where levels of extended working in 2011 were lowest). Adjustment for working status in 2001 in men attenuated the relative risk of extended working in the regions, but the same regions retained significantly higher risk (Model 2). Adjustment for nonemployment‐related socio‐economic status (housing tenure, qualifications, and car ownership) and employment status in 2001 attenuated all significant regional differentials in the relative risk of extended working in men in 2011 (Model 3).

**Table 1:** Relative risk ratios for working in 2011, socio‐economic factors, men aged 40–49 in 2001, England and Wales

		Model 1			Model 2			Model 3		
Variable	*N* = 31,696	RRR	*p*	95% CI	RRR	*p*	95% CI	RRR	*p*	95% CI
Region										
North East (ref)					1			1		
North West		0.99	0.878	0.85–1.14	0.96	0.654	0.82–1.13	0.93	0.386	0.79–1.10
Yorks and Humber		1.06	0.443	0.91–1.24	1.02	0.783	0.86–1.22	1.02	0.867	0.85–1.21
East Midlands		1.42	<0.001	1.20–1.67	1.26	0.014	1.05–1.51	1.20	0.057	0.99–1.44
West Midlands		1.07	0.380	0.92–1.25	0.99	0.938	0.84–1.18	0.95	0.584	0.80–1.13
East of England		1.54	<0.001	1.31–1.80	1.23	0.022	1.03–1.46	1.12	0.226	0.93–1.34
London		1.07	0.391	0.92–1.24	1.07	0.442	0.90–1.26	1.11	0.245	0.93–1.31
South East		1.60	<0.001	1.38–1.86	1.27	0.005	1.08–1.50	1.11	0.208	0.94–1.32
South West		1.59	<0.001	1.35–1.87	1.29	0.006	1.07–1.54	1.16	0.122	0.96–1.39
Wales		0.95	0.560	0.80–1.13	0.95	0.612	0.79–1.15	0.90	0.310	0.74–1.10
Working in 2001					13.25	<0.001	12.26–14.32	8.47	<0.001	7.78–9.21
Number of cars household has access to								1.28	<0.001	1.22–1.33
Highest education qualification										
None								1		
GCSE/O Levels								1.62	<0.001	1.47–1.79
Five O Levels/GCSE								1.49	<0.001	1.34–1.65
Two A Levels/HSC/NVQ3								1.22	<0.001	1.06–1.41
Degree level and above								1.63	<0.001	1.47–1.80
Other								1.31	<0.001	1.16–1.48
Housing tenure										
Owns outright								1		
Mortgage/shared ownership								1.61	<0.001	1.47–1.77
Social renting								0.81	<0.001	1.34–1.65
Private renting								1.28	0.004	1.06–1.41
Rent free								1.26	0.145	1.47–1.80
Other								0.81	0.106	1.16–1.48

*Note*. Authors' own analyses. Data source: Office for National Statistics.

Table 2 shows the same analysis as Table 1, but this time for women only. Women in all regions apart from London and Wales had significantly higher relative risk of extended working compared with the North East: ranging from 1.15× in the North West and West Midlands to 1.6× in the South West. Adjustment for working status in 2001 in women attenuated the relative risk of extended working in these regions, but the same regions remained significantly higher than the North East with the exception of the North West and West Midlands. Additionally, women in London had significantly higher relative risk of extended working (1.2×) once prior working status was taken into account (Model 2). Similar to men, additional adjustment for nonemployment‐related socio‐economic status and employment status in 2001 attenuated regional differentials in the relative risk of extended working in women in 2011. But unlike men, regional differences in extended working remained for the East Midlands, East of England, London, South East, and South West compared with the North East (Model 3).

**Table 2:** Relative risk ratios for working in 2011, socio‐economic factors, women aged 40–49 in 2001, England and Wales

		Model 1			Model 2			Model 3		
Variable	*N* = 33,124	RRR	*p*	95% CI	RRR	*p*	95% CI	RRR	*p*	95% CI
Region										
North East (ref)		1			1			1		
North West		1.15	0.028	1.02–1.31	1.14	0.076	0.99–1.31	1.07	0.335	0.93–1.24
Yorks and Humber		1.19	0.012	1.04–1.36	1.18	0.034	1.01–1.34	1.14	0.090	0.98–1.33
East Midlands		1.34	<0.001	1.17–1.54	1.31	0.001	1.12–1.53	1.25	0.007	1.06–1.46
West Midlands		1.15	0.037	1.01–1.31	1.14	0.081	0.98–1.32	1.11	0.186	0.95–1.29
East of England		1.36	<0.001	1.19–1.55	1.30	0.001	1.12–1.51	1.18	0.033	1.01–1.37
London		1.02	0.755	0.90–1.16	1.17	0.028	1.02–1.35	1.10	0.204	0.95–1.59
South East		1.44	<0.001	1.27–1.63	1.38	<0.001	1.20–1.59	1.20	0.014	1.04–1.38
South West		1.61	<0.001	1.40–1.85	1.56	<0.001	1.34–1.82	1.40	<0.001	1.20–1.64
Wales		1.09	0.242	0.94–1.27	1.10	0.283	0.93–1.30	1.05	0.547	0.89–1.25
Working in 2001					9.18	<0.001	8.66–9.73	7.54	<0.001	7.10–8.01
Housing tenure										
Owns outright								1		
Mortgage/shared ownership								1.54	<0.001	1.42–1.66
Social renting								1.07	0.171	0.97–1.19
Private renting								1.18	0.033	1.01–1.37
Rent free								1.23	0.125	0.95–1.59
Other								1.12	0.441	0.84–1.49
Number of cars household has access to								1.28	<0.001	1.01–1.08
Highest education qualification										
No qualifications								1		
GCSE/O Levels								1.82	<0.001	1.67–1.97
								1.91	<0.001	1.76–2.08
Two A Levels/HSC/NVQ3								2.02	<0.001	1.78–2.30
Degree level and above								2.06	<0.001	1.89–2.25
Other								1.35	<0.001	1.17–1.54

*Note*. Authors' own analyses. Data source: Office for National Statistics.

Table 3 shows the effects of adjustment for health, caring, and demographic characteristics in women only. Prior poor health status (Table 3, Model 1) and providing care (Table 3, Model 2) were also associated with marginal reductions in the significant regional differences in the relative risk of women having extended working lives compared with the unadjusted model. Demographic factors of ethnicity, marital status (including work status of spouse), dependent child(ren) in the household, and age were significantly associated with the relative risk of being in work in women, but had marginal effects on the regional differentials (Table 3, Model 3).

**Table 3:** Relative risk ratios for working in 2011, health, caring, and demographic factors, women aged 40–49 in 2001, England and Wales

		Model 1			Model 2			Model 3		
Variable	*N* = 33,124	RRR	*p*	95% CI	RRR	*p*	95% CI	RRR	*p*	95% CI
Region										
North East (ref)		1								
North West		1.14	0.074	0.99–1.33	1.13	0.084	0.98–1.31	1.13	0.102	0.98–1.31
Yorks and Humber		1.14	0.093	0.98–1.33	1.18	0.034	1.01–1.37	1.16	0.058	0.99–1.35
East Midlands		1.27	0.004	1.08–1.49	1.29	0.001	1.11–1.51	1.30	0.001	1.11–1.52
West Midlands		1.11	0.190	0.95–1.29	1.13	0.100	0.98–1.31	1.15	0.068	0.99–1.34
East of England		1.21	0.013	1.04–1.41	1.29	0.001	1.11–1.49	1.22	0.011	1.05–1.42
London		1.11	0.163	0.96–1.29	1.16	0.041	1.01–1.34	1.21	0.012	1.04–1.41
South East		1.26	0.001	1.09–1.46	1.36	<0.001	1.18–1.56	1.28	0.001	1.11–1.48
South West		1.48	<0.001	1.26–1.74	1.55	<0.001	1.33–1.81	1.49	<0.001	1.28–1.75
Wales		1.08	0.375	0.91–1.29	1.11	0.236	0.94–1.31	1.08	0.393	0.91–1.28
Working in 2001		6.79	<0.001	6.39–7.22	8.88	<0.001	8.37–9.41	9.77	<0.001	9.17–1.41
Limiting long‐term illness		0.48	<0.001	0.79–1.03						
Health										
Good health		1								
Fair health		0.67	<0.001	0.63–0.72						
Poor health		0.44	<0.001	0.39–0.50						
Caring for others										
No care provided					1					
1–19 hr					0.92	0.068	0.85–1.01			
20–49 hr					0.72	<0.001	0.60–0.86			
50+ hr					0.56	<0.001	0.48–0.64			
Marital status										
No spouse								1		
Spouse working								1.05	0.169	0.98–1.13
Spouse not working								0.75	<0.001	0.66–0.85
Spouse sick								0.50	<0.001	0.43–0.59
Dependent children in household										
None								1		
Youngest aged 0–4								1.89	<0.001	1.67–2.15
Youngest aged 5–11								1.81	<0.001	1.67–1.96
Youngest aged 12–15								1.50	<0.001	1.38–1.63
Youngest aged 15–18								1.25	<0.001	1.13–1.39
Ethnicity										
White								1		
Mixed								0.58	0.001	0.43–0.80
Asian								0.51	<0.001	0.45–0.57
Black								1.27	0.039	1.01–1.60
Other								0.79	0.196	0.56–1.13
Age single years								0.92	<0.001	0.91–0.92

*Note*. Analysis authors own. Data source: Office for National Statistics.

Demographic factors of ethnicity, marital status (including work status of spouse), dependent child(ren) in the household, and age were significantly associated with the relative risk of being in work in men, but had marginal effects on the regional differentials. Prior poor health status and providing care were also associated with marginal reductions in the significant regional differences in the relative risk of men having extended working lives compared with the unadjusted model (results for these two analyses are not shown).

Table 4 showed the additional effects of workplace in (women only) on attenuation on regional inequalities in remaining in work. Prior employment characteristics were important for explaining regional variation in extended working in a subset of women who were employed in 2001, with women employed in lower skilled jobs less likely to remain in work (Table 4). Women employed by larger employers in 2001 had lower relative risk of remaining in work as did those working at distances of 200 km or more from home or abroad whereas access to cars increased the chance of remaining in work; the higher the hours worked in 2011, the more likely to be in work in 2011. Adjustment for these factors in addition to health status, provision of care, and demographic factors attenuated all regional variations between the North East and the other regions apart from the South West where women had 1.25× higher risk of extended working in the fully adjusted model.

**Table 4:** Relative risk ratios for working in 2011, employment factors and full model, women in work aged 40–49 in 2001, England and Wales

*N* = 25,008	RRR	*p*	95% CI
Region			
North East (ref)	1		
North West	1.06	0.552	0.88–1.28
Yorks and Humber	1.12	0.252	0.92–1.37
East Midlands	1.20	0.080	0.98–1.47
West Midlands	1.12	0.268	0.92–1.36
East of England	1.07	0.511	0.88–1.30
London	1.04	0.731	0.85–1.26
South East	1.08	0.420	0.90–1.30
South West	1.26	0.024	1.03–1.54
Wales	1.07	0.550	0.86–1.34
Size of workplace			
1–9	1		
10–24	1.12	0.057	1.00–1.26
25–499	1.21	<0.001	1.10–1.34
500+	1.15	0.022	1.02–1.29
Hours of work in 2001	1.01	<0.001	1.01–1.02
Employment type			
Nonmanual	1		
Manual	0.85	<0.001	0.78–0.93
Otherwise classified	0.70	0.153	0.44–1.14
Distance travelled to workplace			
0–49 km			
50–99 km	0.95	0.342	0.86–1.05
100–199 km	0.95	0.371	0.84–1.07
200+ km	0.81	0.006	0.70–0.94
Outside of United Kingdom	0.76	0.037	0.59–0.98
At home	0.87	0.055	0.76–1.00
Has a limiting long‐term illness	0.63	<0.001	0.55–0.72
Health			
Good health	1		
Fair health	0.75	<0.001	0.68–0.81
Poor health	0.42	<0.001	0.36–0.50
Caring for others			
No care provided	1		
1–19 hr	0.98	0.732	0.88–1.09
20–49 hr	0.86	0.269	0.66–1.12
50+ hrs	0.69	0.004	0.54–0.89
Housing tenure			
Owns outright	1		
Mortgage/shared ownership	1.24	<0.001	1.13–1.37
Social renting	0.98	0.820	0.85–1.14
Private renting	1.09	0.440	0.88–1.36
Rent free	1.31	0.199	0.87–1.98
Other	1.00	0.987	0.66–1.51
Number of cars household can access	1.07	0.004	1.02–1.12
Highest education qualification			
No qualifications	1		
GCSE/O Levels	1.25	<0.001	1.11–1.40
Five O Levels/GCSE	1.31	<0.001	1.17–1.47
Two A Levels/HSC/NVQ3	1.36	<0.001	1.15–1.61
Degree level and above	1.27	<0.001	1.12–1.43
Other	1.12	0.208	0.94–1.33
Ethnicity			
White	1		
Mixed	0.73	0.184	0.46–1.16
Asian	0.70	<0.001	0.59–0.84
Black	1.52	0.018	1.07–2.16
Other	0.81	0.416	0.49–1.34
Marital status			
No spouse	1		
Spouse working	0.85	0.002	0.77–0.94
Spouse not working	0.77	0.010	0.63–0.94
Spouse sick	0.70	0.006	0.55–0.91
Dependent children in household			
None	1		
Youngest aged 0–4	1.44	<0.001	1.17–1.76
Youngest aged 5–11	1.80	<0.001	1.61–2.02
Youngest aged 12–15	1.42	<0.001	1.28–1.58
Youngest aged 15–18	1.25	<0.001	1.10–1.42
Age single years	0.91	<0.001	0.90–0.92

*Note*. Author's own analysis. Data source: Office for National Statistics.

**Figure 2:**
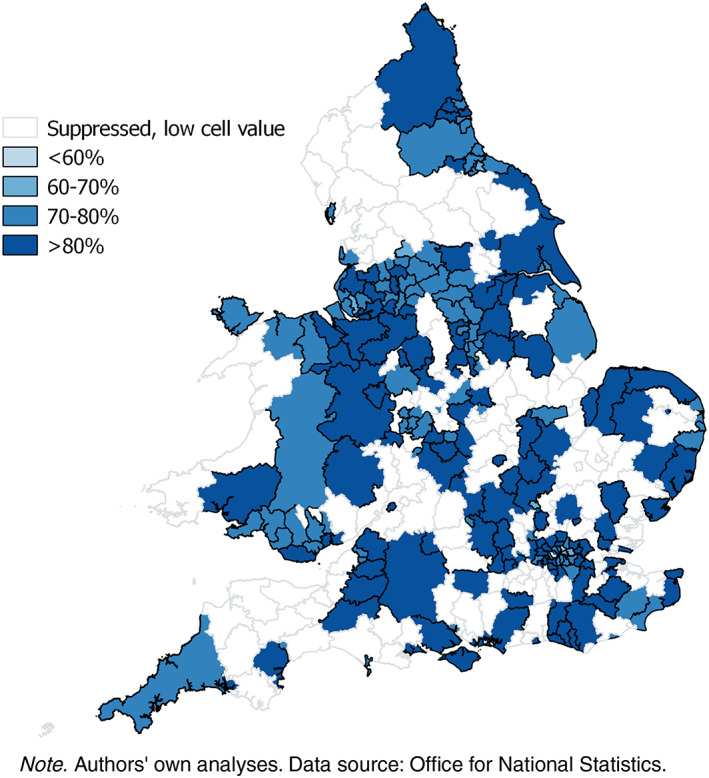
Proportion of men aged 50–59 in paid work in 2011 by local authority, England and Wales

**Figure 3:**
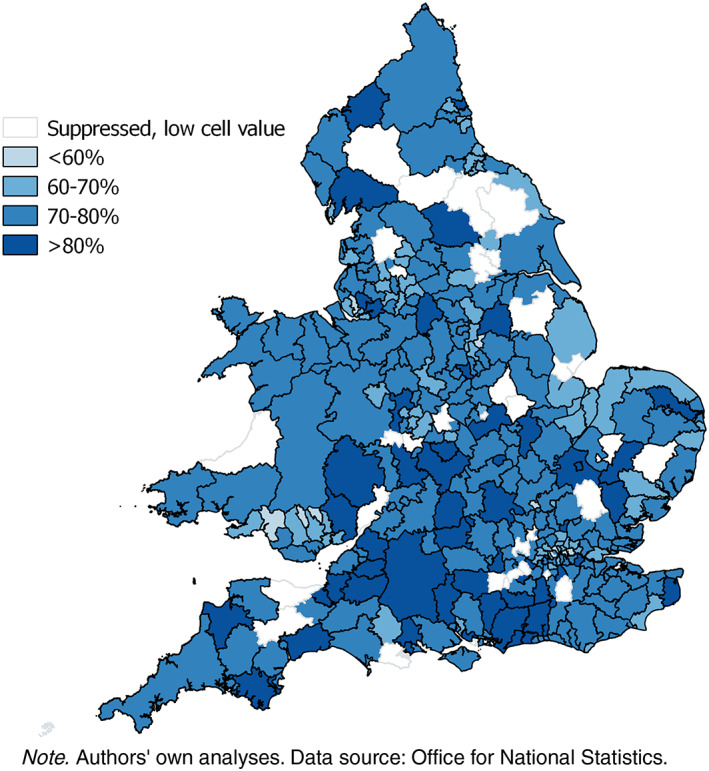
Proportion of women aged 50–59 in paid work in 2011 by local authority, England and Wales

Lower level geographies of extended working are shown in Figures 2 and 3 with the geography of extended working in 2011 to age 50–59 by local authority illustrating the regional divide especially in women. The missing data are where either the cell count for the proportion of working or nonworking adults or the overall sample size is below 10.

## 5 DISCUSSION

In this nationally representative study, there were large regional inequalities in extended working with up to 1.6 times greater risk of working beyond age 50 to retirement age in the south of England compared with the North East. Although these associations were somewhat attenuated by adjusting for prior working status, inequalities remained. For men, it was indicators of higher socio‐economic status, including qualifications and access to cars as well as being in higher socio‐economic classes, which explained the regional variation in men's extended working lives. Financial imperatives may also have driven extended working with those still holding a mortgage or living in the private rental sector more likely to be in work.

Similarly for women, adjustment for socio‐economic factors reduced regional inequalities for extended working, but women in the South and East of England still had significant substantially higher relative risk of extended working than women in the North East. Additionally, prior employment characteristics were important for explaining regional variation in extended working in women, with women employed in lower skilled jobs less likely to remain in work. Those employed by larger employers in 2001 had lower relative risk of remaining in work possibly due to formal compulsory corporate retirement ages (prior to legislation largely preventing these from 2011) and potentially more adequate pension provision. Higher hours of work in 2001 were associated with higher chances of being in work in 2011—possibly those in less than full‐time work in 2011 were making a transition to retirement. Travel to work may be a barrier to EWL—access to cars increased the chance of remaining in work, and longer distance to previous employer (200 km and above) in 2001 reduced the chance of being in work in 2011. Although demographic, health, and caring factors were also significantly associated with the relative risk of being in work for women, they did not have significant effects on the regional differentials, which is counter to the national level.

Though much of the regional differentials can be explained by the characteristics of the population of these regions, that is, the factors are compositional rather than contextual, the largest single factor associated with extended work is prior employment and that is likely to be due to contextual factors. Policies that do not address life course issues such as low levels of education and high levels of unskilled employment will only be partially successful in enabling people to remain in work longer. Indeed, it is those that least need to remain in work due to likely lifelong higher salaries having higher levels of education and social class that are most able to do so. This is particularly an issue for women. In 2011, 26.5% of the adult population (age 16 and over) had no formal qualifications in the North East region compared with only 17.6% in London (ONS, 2018). Addressing regional inequalities in extended working will need policies that increase skills and education in later life rather than simply targeting those “receptive” to extending working. The IPPR North's recommendation of “life course work centres” of programmes to support businesses and individuals may be relevant here (Round, 2016).

The subset analysis is restricted to those in work in 2001 to include characteristics of workplace in 2001 and may bias our results, given that some women in the sample were in their 40s in 2001 and may have been on career breaks for childcare and so forth. Ten percent of those working in 2011 who were not working at the time of the 2001 census but had worked between 1991 and 2001 compared with only 1% of those working in 2011 had last worked between 1981 and 1991, suggesting that there is an influence of prior recent working status. Status change such as transition to/from caring or marital status change may be important and will be considered in future analysis but is beyond the scope of this paper.

### 5.1 Policy recommendations

Education and training across the life course will be key to increasing extended working lives with prior employment, skill level of most recent job, and educational qualifications all being major factors associated with regional variations in working up to and beyond state retirement age. Policy should be developed with lifelong education and training for both workers and the unemployed of all ages in qualifications and skills that will enable them to remain in the workforce by looking at the occupations of those currently employed in later life and the national and regional skills shortage.

The Pensions Advisory Service (2018) suggests that retirement offers the chance to set up their own businesses and quotes figures of “32% of those working beyond state pension age are self‐employed, compared with just 13% of younger workers.” It does not include a source nor indicate what proportion has always been self‐employed and what proportion is newly self‐employed. Analysis of the self‐employed occupations of older workers and the national and regional skills shortages and the feasibility of training other older workers in these occupations should be considered with funding and training to support older workers into self‐employment.

As both long distances to place of employment and lack of access to car are associated with lower relative risk of and regional differences in remaining in work for older women, investigation of the type of work that is affected is required and investment in training and technology may be required to support flexible working either at alternative sites or from home.

This paper has focused on those not yet reaching state pension age. Disparity also exists in the regional employment rates of adults aged 65 and over partly due to regional variation in healthy ageing. As state pension ages increase to 68 in 2046 for larger number of people, it is working at age over 60 that will be crucial in providing later life income (Department for Work and Pensions, 2017). The North East has the smallest gender gap in employment in the 65 and over age group, but that is due to the lowest male employment rates not advantageous rates for women (Government Office for Science, 2016). Older workless couples not yet eligible for pensions will become a growing concern, and this must be monitored and considered in service provision.

Returning to the Department for Work and Pensions' (2012) descriptors of “high risk/vulnerable groups” or “more receptive groups” for extended working, this paper does not support that there are employment sectors, geographical regions, or locales where there were concentrations of “high resistance” to extended working life, but rather regions where previous employment and education facilitate or limit late life employment. Policies must be developed that remove the rhetoric around “resistance” and support those not in work to draw on opportunities for EWL.
